# The First Report of *mcr-1*-Carrying *Escherichia coli* Originating from Animals in Serbia

**DOI:** 10.3390/antibiotics10091063

**Published:** 2021-09-03

**Authors:** Dušan Mišić, Ferenc Kiskaroly, Michael P. Szostak, Adriana Cabal, Werner Ruppitsch, Tanja Bernreiter-Hofer, Viktoria Milovanovic, Andrea T. Feßler, Franz Allerberger, Joachim Spergser, Elke Müller, Stefan Schwarz, Sascha D. Braun, Stefan Monecke, Ralf Ehricht, Maciej Korus, Damir Benković, Malgorzata Korzeniowska, Igor Loncaric

**Affiliations:** 1 Department of Functional Food Products Development, Faculty of Biotechnology and Food Science, Wroclaw University of Environmental and Life Sciences, 51-630 Wrocław, Poland; maciej.korus@upwr.edu.pl (M.K.); malgorzata.korzeniowska@upwr.edu.pl (M.K.); 2 Department of Bacteriology, Veterinary Specialistic Institute “Subotica”, 24000 Subotica, Serbia; ferenc.kiskarolj@vsisu.co.rs; 3 Institute of Microbiology, University of Veterinary Medicine, 1010 Vienna, Austria; michael.szostak@vetmeduni.ac.at (M.P.S.); tanja.bernreiter-hofer@vetmeduni.ac.at (T.B.-H.); 00609827@students.vetmeduni.ac.at (V.M.); joachim.spergser@vetmeduni.ac.at (J.S.); Igor.Loncaric@vetmeduni.ac.at (I.L.); 4 Austrian Agency for Health and Food Safety (AGES), Institute of Medical Microbiology and Hygiene, 1090 Vienna, Austria; adriana.cabal-rosel@ages.at (A.C.); werner.ruppitsch@ages.at (W.R.); franz.allerberger@ages.at (F.A.); 5 Department for Farm Animals and Veterinary Public Health, University Clinic for Swine, University of Veterinary Medicine, 1210 Vienna, Austria; 6 Centre for Infection Medicine, Department of Veterinary Medicine, Institute of Microbiology and Epizootics, Freie Universität Berlin, 14163 Berlin, Germany; andrea.fessler@fu-berlin.de (A.T.F.); stefan.schwarz@fu-berlin.de (S.S.); 7 Leibniz Institute of Photonic Technology (IPHT), 07745 Jena, Germany; elke.mueller@leibniz-ipht.de (E.M.); sascha.braun@leibniz-ipht.de (S.D.B.); stefan.monecke@leibniz-ipht.de (S.M.); Ralf.Ehricht@leibniz-ipht.de (R.E.); 8 InfectoGnostics Research Campus, 07743 Jena, Germany; 9 Institute for Medical Microbiology and Virology, Dresden University Hospital, 01307 Dresden, Germany; 10 Institute of Physical Chemistry, Friedrich Schiller University Jena, 07743 Jena, Germany; 11 Department of Molecular Diagnostics, Veterinary Specialized Institute “Sombor”, 25000 Sombor, Serbia; vsi.sombor@gmail.com

**Keywords:** colistin, *mcr*, resistance, *E. coli*, turkeys, biocides

## Abstract

The aim of this study was continuous monitoring of the presence of *mcr-1* to *mcr-5* genes in *Enterobacterales* isolated from cattle, pigs, and domestic poultry at intensive breeding facilities in Northern Vojvodina, Serbia, from 1 January 1 to 1 October 2020. Out of 2167 examined samples, *mcr-1* was observed in five *E. coli* isolates originating from healthy turkeys. Four isolates belonged to the phylogenetic group B1, and one isolate to the phylogenetic group A. Detected *E. coli* serogenotypes (somatic O and flagellar H antigens) were O8:H25 and O29:H25. Core-genome multi-locus sequence typing (cgMLST) revealed three ST58 isolates clustering together in Clonal Complex (CC) 155 and two singletons of ST641-CC86 and ST410-CC23, respectively. Clonotyping revealed CH4-32 (*n* = 3), CH6-53 (*n* = 1) and CH4-24 (*n* = 1). In all isolates, the *mcr-1* gene was located on a large IncX4 replicon type plasmid. Eight virulence-associated genes (VAGs) typical of avian pathogenic *E. coli* (APEC) (*fyuA, fimH*, *hlyF*, *iss*, *ompT*, *sitA*, *traT*, *iroN*) were detected in four isolates. These isolates were investigated for susceptibility to four biocides and revealed MIC values of 0.125% for glutardialdehyde, of 0.00003–0.00006% for chlorohexidine, of 4–6% for isopropanol and of 0.001–0.002% for benzalkonium chloride. All obtained MIC values of the tested biocides were comparable to the reference strain, with no indication of possible resistance. This is the first report of *mcr-1.1*-carrying *E. coli* from Serbia. Although only samples from turkeys were *mcr*-positive in this study, continuous monitoring of livestock samples is advised to prevent a spill-over from animals to humans.

## 1. Introduction

Colistin (polymyxin E) was introduced into medical and veterinary clinical practice in the early 1950s [1] but due to its high toxicity, systemic administration of colistin in humans was restricted for decades [2]. However, the emergence of Gram-negative bacteria resistant to critically important antibiotics for humans, especially to carbapenems, led to the gradual reintroduction of colistin into human medicine as a last resort antibiotic for the systemic treatment of severe nosocomial infections [1,2,3,4]. In contrast to human medicine, colistin has been widely used in large quantities in animals on all continents contributing to the emergence of resistance [1,2].

Colistin resistance may be mediated by chromosomal mutations or encoded by transferable, plasmid-mediated *mcr* genes (*mcr-1* to *mcr-10*) [2,5]. Retrospective studies have shown that the *mcr-1* gene was present in bacteria that originated from poultry in China as early as in the 1980s, and that the increased *mcr* prevalence from 2015 coincided with the intensification of epidemiological studies of this topic [6,7]. The *mcr* genes were found in bacteria isolated from humans, pigs, cattle, veal calves, broilers, chicken, turkeys, dogs, cats, food and water specimens from 57 countries, including 19 countries from Europe [1,7]. The *mcr* variants were mostlty found in *E. coli* and different *Salmonella* serovars, but also in *Klebsiella pneumoniae*, *Shigella sonnei*, *Enterobacter aerogenes*, *Enterobacter cloacae*, members of *Cronobacter*, *Kluyvera*, *Citrobacter*, *Providencia, Raoultella* and *Moraxella* genera [1,2,3,4,5,6,7,8].

In comparison to isolates of human origin, the prevalence of *mcr* in bacteria from animals and food seems to be higher. Moreover, the co-occurrence with florfenicol resistance, which is a purely veterinary antibiotic, indicates that colistin resistance mediated by *mcr* genes may be transmitted from animals to humans or even via the food chain [6,8,9,10,11,12]. Consequently, the European Medicines Agency (EMA) and World Health Organization (WHO) re-categorized colistin as “restricted” for medical and veterinary use due to its potential risk for Public Health [1,13,14,15]. However, there are countries in which the use of colistin is still approved as a growth promoter in food animals. This includes nine countries from Latin America, Asia, Africa and Oceania [15,16,17].

Although colistin-resistant *Enterobacterales* have been previously found in both humans and animals in Serbia [18,19], the associated resistance mechanisms have been linked to chromosomal mutations, and no *mcr* genes have been detected so far. Concern among the scientific and medical community that the efficacy of colistin could be lost due to the rapid spread of the *mcr* genes, and the continuous emergence of new *mcr* alleles, requires further research.

## 2. Results

Colistin-resistant isolates were neither found in the chicken, hen and cattle samples nor in those from pigs. However, five different colistin-resistant *E. coli* isolates (isolate IDs: 51, 52, 15/2, 16/2, and 72/2) were detected. These isolates had been obtained in June 2020 from intestines of turkeys from the same farm. The turkeys were 12-weeks old, clinically healthy and originated from incubator stations in Croatia (imported as one day old chicks). Antibiotic susceptibility testing was not performed during the quarantine of the animals. Isolates displayed colistin MICs of 2 µg/mL (51, 15/2, 16/2) and 8 µg/mL (52, 72/2) and all carried the *mcr-1.1* gene. The turkeys from which the isolates were obtained had not been treated with antibiotics before the time of the sampling and farmers denied having used colistin on the birds. According to mlplasmids analyses, the *mcr-1.1* gene might be located on plasmids.

Four of the five isolates (51, 15/2, 16/2 and 72/2) were resistant to at least one antimicrobial agent from three different classes and thus categorized as multidrug-resistant (MDR) (Table 1.).

Isolate 72/2 was resistant to antibiotics from 6 different classes. Solely isolate 52 was resistant to only ampicillin and colistin. All isolates carried the *bla*_TEM-1_ gene and were resistant to ampicillin. Tetracycline resistance encoded by the gene *tet*(A) was detected in four isolates, except for isolate 72/2, in which the tetracycline resistance genes *tet*(A) and *tet*(M) were detected. Resistance to ciprofloxacin and the presence of the plasmid-mediated quinolone resistance (PMQR) gene *qnrS1* was found in three isolates. One isolate carried the *qnrS1* gene. Mutations in the quinolone resistance-determining region (QRDR) of *gyrA* and/or *parC* were also found. Within the GyrA region, Ser83Leu substitutions were detected in four isolates. In isolate 72/2, an Asp87Asn substitution in GyrA and a Ser80Ile substitution in ParC were found. In addition, resistance to the combination trimethoprim and sulfamethoxazole, chloramphenicol and florfenicol, and the related antimicrobial resistance genes (ARGs) *sul2*, *sul3*, *dfrA12*, and *cmlA1* were found in isolate 72/2. The obviously inactive genes *aadA1* and *aadA2* were detected in isolate 72/2, while *strA* and *strB* genes were found in isolate 51.

Isolates 51, 52, 15/2 and 16/2 belonged to the phylogenetic group B1 and isolate 72 to the phylogenetic group A (Table 1). Detected *E. coli* serogenotypes (somatic O and flagellar H antigens) were O8:H25 and O29:H25. Sequence types (STs), that were extracted from the Whole Genome Sequencing (WGS) data, belonged to ST 58 (15/2, 16/2, 51) and clonal complex (CC) 155. WGS-based core genome MLST (cgMLST) revealed that these three isolates clustered together. The other two isolates 52 and 72/2 represented the singletones ST641-CC86 and ST410-CC23, respectively. Clonotyping revealed CH4-32 (*n*-3), CH6-53 (*n* = 1) and CH4-24 (*n* = 1). Taken together, *mcr-1.1*-carrying E. coli isolates belonged to three different clones: B1-O8:H25-ST58-CH4-32 (isolates 51, 15/2, 16/2), B1-O29:H25-ST641-CH6-53 (isolate 52) and A-O8:H25-ST410-CH4-24 (isolate 72/2). They shared between 96.18 and 100% DNA similarity with corresponding reference sequences.

**Figure 1:**
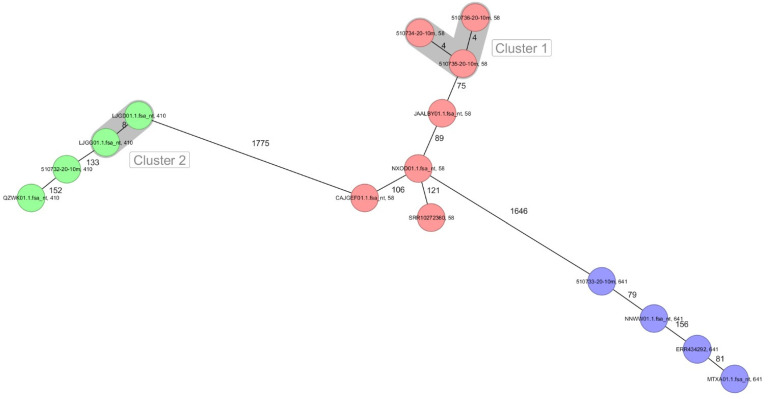
Minimum spanning tree for five Serbian *mcr-1*-carrying *E. coli* and close related isolates based on the cgMLST of *E. coli*. Colors correspond to the sequence type. Each circle represents isolates with an allelic profile based on the sequence of 2513 core genome targets. Numbers refer to the allelic differences between two isolates. Isolates with closely related genotypes were identified with a maximum of 10 allelic differences and are shaded in grey.

Of the 10 selected NCBI *E. coli* genomes belonging to different sources and included in the genomic comparison, none of them clustered together with any of our isolates. The closest isolate of ST410 to our isolates differed by 133 or more alleles (LJGG01.1). Regarding ST58, the closest isolate presented more than 75 or more allelic differences (JAALBY01.1) with our isolates and for ST641, at least 79 or more allelic differences were seen between an NCBI isolates (NNWW01.1) and our ST641 isolates (Figure 1).

In total, six different plasmids, IncFIB(AP001918) (isolates 15/2, 16/2, 51, 72/2), IncFII(pRSB107) (isolates 15/2, 16/2, 51), IncX1 (all isolates), IncX4 (all isolates), IncFII (isolate 72/2), and p0111 (isolate 72/2) were identified.

The presence of eight virulence-associated genes (VAGs) (*fyuA*, *fimH*, *hlyF*, *iss*, *ompT*, *sitA*, *traT*, *iroN*) typical of avian pathogenic *E. coli* (APEC) was detected in the isolates belonging to B1-O8:H25-ST155-CH4-32 and B1-O29:H25-ST641-CH6-53). We also detected the uropathogenic *E. coli* (UPEC) *fyuA* gene in the same isolates. A list of all detected VAGs is shown in Table 2.

Biocide susceptibility testing revealed, that all *mcr-1*-carrying *E. coli* isolates, except isolate 72/2, had benzalkonium chloride (BAC) MIC values of 0.001%. The remaining isolate had a MIC value for BAC of 0.002%. Chlorhexidine (CHX) MIC values for three isolates (51, 15/2, 16/2) were 0.00006% and for the remaining two isolates (52 and 72/2) 0.00003%. MIC values for isopropanol were 4% for isolates 51, 16/2, 72/2 and 6% for isolates 52 and 15/2. All tested isolates showed a glutaraldehyde (GDH) MIC value of 0.125% (Table 3). The obtained MIC values for all biocides had comparable -MIC ranges to *E. coli* ATCC^®^ 10536.

## 3. Discussion

Our work presents the results of a continuous monitoring study on phenotypic colistin resistance and the presence of *mcr-1* to *mcr-5* genes in *Enterobacterales* isolated from cattle, pigs, and domestic poultry (turkeys, laying hens, broilers) at intensive breeding facilities in Northern Vojvodina, Serbia, from 1 January to 1 October 2020.

A total of five *mcr-1.1*-carrying *E. coli* isolates were obtained from turkeys in June 2020. According to the local authorities, the turkeys of that farm had not been treated with antibiotics, including colistin. The origin of these isolates remains unclear, since it was not possible to prove an epidemiological link with Croatia (the origin of the respective turkey chicks), especially when considering that *mcr* genes have not yet been reported in Croatia, neither in humans nor in animals. Moreover, samples were taken again in September 2020 at the same farm from newly imported turkey chicks, as well as from farm workers, but neither an *mcr* gene nor phenotypically colistin-resistant isolates were detected. Likewise, *E. coli* clones belonging to ST640, ST58 and ST410 have not been reported in Croatia so far, neither in humans nor in animals. However, chromosomal colistin resistance due to mutations has been previously detected in Croatia [20,21,22].

Despite many published papers on the finding of *mcr-1* genes in bacteria originating from humans, animals, food, and the environment, it is still difficult to gain accurate insight into the distribution and epidemiology of *mcr*-carrying bacteria of animal origin. Large and comprehensive epidemiological and surveillance studies involving different animal species are still rare, and most publications deal with reports on single isolates [23,24,25,26,27,28,29].

The hypothesis that colistin resistance encoded by *mcr* genes is most often transmitted from animals to humans is mostly based on the findings of a higher prevalence of *mcr*-carrying bacteria in animals than in humans [1,6,7,8,11,30]. Another hypothesis is that there is a positive correlation between colistin resistance and colistin usage rates in animals [1,30,31]. The latter is well-founded by data from countries where colistin is not used in animals or its use is minimal. For instance, in Iceland, Norway, Finland and Denmark, where the sale of colistin quantities is negligible or non-existent, no *mcr* gene has been reported in bacteria obtained from animal sources or, when *mcr* was found, it was more commonly detected in imported animals or imported meat products [7,31,32]. This observation is similar in the USA, where colistin is not approved for use in food-producing animals, and so far, only two *mcr-1*-carrying *E. coli* isolates have been reported from pigs in an intensive breeding farm [33] and one from a pork meat sample [34]. The same hypothesis is supported by the data on the enormous consumption of colistin in China. China is the largest user of colistin in animals, and so far, the largest number of *mcr*-carrying isolates (>2500) have been found in samples from animals in that country [7]. Although a positive correlation between the consumption of colistin and the prevalence of the *mcr* gene has been demonstrated, the distribution of colistin-resistant and/or *mcr-*carrying strains may be influenced by other factors. A retrospective study including 250 *E. coli* isolated from health pigs between 2004 and 2007 in Japan, where the use of colistin is allowed not only in food-producing animals but also as growth promoter, reported the absence of colistin-resistant *E. coli* isolates [35]. In contrast, another report showed a positive association between the use of colistin in pigs and the occurrence of 13% of *mcr-1*-carrying pathogenic *E. coli* in pigs in that country [36]. Whether or not the positive association between the consumption and the occurrence of resistance to colistin has been proved, there are indications that may support this. The spread of *mcr*, except direct selection is also influenced by cross-selection, co-selection (consumption of antibiotics other than colistin), unknown biological mechanisms, negative fitness of bacteria carrying *mcr* genes, clonal spreading, insects, age of the animals, pathogenic or commensal microorganism as well as the type of samples—usually the highest prevalence of mcr was found in pathogenic bacteria originated from young animals and fecal samples compared to indicator *E. coli* isolated from swabs or intestinal contents [35,37,38,39].

Results of a large number of studies have shown that the prevalence of the *mcr-1* gene tends to be higher in *E. coli* originating from turkeys, regardless of the colistin usage rate. However, this should not be considered as a general rule, as other factors might be associated, including geographical variation. In France, for instance, the prevalence of *mcr-1*-carrying *E. coli* reported between 2011 and 2014 was significantly higher in turkeys (5.9%) than in broilers (1.6–1.8%) and in pigs (<1%) [40]. Germany, one of the largest consumers of colistin in veterinary medicine in the EU, but also the largest pig producer in Europe, the prevalence of *mcr*-carrying *E. coli* seemed to be higher in turkeys than in pigs [39,41]. According to Irrgang et al. [38], the detected prevalence of *mcr-1*-carrying *E. coli* originating from turkeys in Germany was 10.2% in 2010, 17.9% in 2011, 11.7% in 2012 and 10.4% in 2014. The same source stated that the prevalence of *mcr-1*-carrying *E. coli* in pigs in 2011 and 2015 was 1.5%. Another retrospective study [28] stated that of a total of 577 isolates collected from humans, animals, and food in Germany between 2009 and 2016, including a total of 424 *E. coli* from livestock, only three *mcr-1-*carrying *E. coli* originated from pigs. A slightly higher prevalence of 9.9% *mcr-1*-carrying *E. coli* in pigs was detected in a German retrospective study from 2011–2012 [39]. However, such findings should be taken with caution, since the isolates originated from pooled fecal samples that were pre-selected by growth on selective media supplemented with cefotaxime and enrofloxacin.

Italy and Spain were considered countries where the use of polymyxins in animal production was the highest in the EU [1,31], coinciding also with a high prevalence of *mcr* genes in *E. coli* from animal origin including turkeys. In Italy, 19.3% of *mcr-1*-carrying *E. coli* was detected in turkeys in 2013 [1], increasing to 22.9% and 25.9% in 2014 and 2015, respectively [1,42]. During the same period, the prevalence of *mcr-1*-carrying *E. coli* in fattening pigs was reported to be 0.6–6.5% [43]. In another Italian study, about 75% (*n* = 51) of the *E. coli* from diarrheal pigs retrieved during 2015–2016 were *mcr-1*-positive [44]. Regarding wildlife, other authors in Italy have reported 44.6% (*n* = 168) of the *E. coli* isolated from haunted wild boars as either *mcr-1*- or *mcr-2*-positive, which was the basis to hypothesize that wild boars may be a reservoir of colistin-resistant isolates [45].

In Serbia, colistin resistance has already been reported in Gram-negative bacteria isolated from animals and humans, but the mechanisms have been always linked to chromosomal mutations [18,19,46]. Based on data of the Agency for Medicinal Products and Medical Devices of the Republic of Serbia [47], consumption of colistin in veterinary medicine has been continuously declining in Serbia since 2011, with the most noticeable decline occurring in the period from 2016 to 2018. Unfortunately, there are no official data on the usage of colistin in animal production for the years 2019 and 2020 and hence, comparisons with other countries are not possible. However, the use of antibiotics as a growth promoter, including colistin, has been officially banned in Serbia since 2010.

As shown in retrospective studies, *E. coli* strains of ST410, ST58 and ST641 carrying the *mcr-1* gene are present in animals across Europe since 2004 [48,49,50]. All *E. coli* clonal complexes found in our study (ST58, ST410 and ST641) have so far been associated with extraintestinal infections in humans and animals. *E. coli* ST410 has been found many times in humans (2002 in Canada, 2008 and 2010 in Spain, 2008 in Brazil, 2010 and 2014 in Norway) as well as in animals (2004 in cats, Germany; 2005 in dogs, Germany; 2009 in dogs, France; 2007 in turkeys, Germany; 2014 in pigs Thailand). *E. coli* ST641 has been reported from diseased humans (2007, Canada; 2009, Africa; 2016, USA), pigs (2004, Germany; 2011, Portugal; 2014, Thailand), fish (2001, Portugal), wild boars (2011 and 2015, Denmark), celery (2003, USA), beef (2016, USA) and chickens (2014, 2015, 2016, USA). *E. coli* ST58 has been detected in humans (2011, USA), chickens (2016, Africa), Cattle (2010, Ireland; 2018, 2019, USA), pigs (2005, Germany; 2009, China; 2019, USA), turkeys (2006, Germany; 2018, Netherlands) and dogs (2009, Netherlands) (https://enterobase.warwick.ac.uk/species/index/ecoli, accessed on 23 June 2021) [49].

Some of the genes encoding virulence factors (VFs) found in *mcr-1* carrying isolates in this study, (*fyuA*, *fimH*, *hlyF*, *iss*, *ompT*, *sitA*, *traT*, *iroN*) are typical for extraintestinal pathogenic *E. coli* (ExPEC) and its subcategories-uropathogenic *E. coli* (UPEC) and avian pathogenic *E. coli* (APEC) [50]. *FimH* adhesins/type 1 fimbriae, are important for successful colonization of UPEC on the urinary bladder epithelium but it is also referred as a major virulence factor of *E. coli* belonging to APEC. At the same time, *fimH*, *fyuA*, *hly*, *ompT* and *iroN* are usually found in enterohaemorrhagic *E. coli* (EHEC) isolated from both humans and animals [50]. Finding of those VFs is not sufficient for our strains to be categorized in any of the ExPEC categories, but because they were located in *E. coli* belonging to ST155 and ST641 which both were previously found in diseased humans and animals, pathogenic potential and possible risk for Public Health is indicated.

There is not much information in the scientific literature on the epidemiology and significance of *E. coli* O8:H25. *E. coli* belonged to this serotype has recently been isolated from chicken breast and beef samples in Brazil. Isolate possessed intimin but it was non-toxin producing, and was classified as atypical Enteropathogenic *E. coli* (aEPEC) [51].

*E. coli* O29:H25 serotype, to our knowledge, has not been reported to this date and this is the first report of this serogenotype with the significance in veterinary medicine. *E. coli* O29 with another flagellar antigen type have been reported as *shiga*-toxin producing (enteroinvasive) strain isolated from humans [52]. Similarly, the H25 *E. coli* of a different somatic O antigenic structure was also found in *shiga* toxin-producing isolates [53].

Biocides are used to control infectious diseases on poultry farms and in slaughterhouses and can contribute to the emergence and spread of bacteria resistant to antibiotics [54,55]. In addition, resistance to biocides has already been reported. In our study, we wanted to test whether *mcr-1* carrying *E. coli* isolates were sensitive to biocides widely used in animal production and veterinary medicine. Obtained MIC values of glutardialdehyde, isopropanol, benzalkonium chloride and chlorhexidine were unimodal, in acceptable ranges, equal to ranges previously established for *E. coli* ATCC^®^ 10536.

## 4. Materials and Methods

The isolates were collected from 1 January 2020, to 1 October 2020, and included samples obtained in routine microbiological diagnostics at the Veterinary Specialist Institute in Subotica. A total of 2167 samples from intensive breeding farms in Northern Vojvodina, Serbia were processed, of which 1174 were from broilers, 502 from cattle, 332 from laying hens, 128 from pigs and 29 from turkeys. Specimens included feces, skin, nasal and rectal swabs and internal organs of dead animals. Intestinal samples taken from poultry, originated from healthy sacrificed birds as part of the routine control for the presence of *Salmonella*. Isolation of microorganisms and primary screening for colistin resistance were performed in the microbiological laboratory of the Veterinary Specialist Institute in Subotica. All samples were inoculated on MacConkey agar (Becton Dickinson, Heidelberg, Germany) supplemented with 2 µg/mL colistin (Carl Roth, Karlsruhe, Germany) [56]. Colonies selected from these MacConkey agar plates were identified to the species level by matrix-assisted laser desorption/ionization-time-of-flight (MALDI-TOF) mass spectrometry (Bruker Daltonik, Heidelberg, Germany). Colistin MICs (0.125–256 µg/mL) were established with broth microdilution according to standards of the Clinical and Laboratory Standards Institute (CLSI) [57]. Isolates that displayed colistin MICs of ≥2 µg/mL were further characterized following a previously described multiplex-PCR protocol for the detection of the *mcr*-1 to -5 genes [58].

The *mcr*-*1*-carrying isolates were further tested for their antimicrobial susceptibility by agar disk diffusion according to CLSI [57]. The following disks were used: ampicillin (10 µg), cefotaxime (30 µg), ceftazidime (30 µg), cefoxitin (30 µg), aztreonam (30 µg), imipenem (10 µg), meropenem (10 µg), gentamicin (10 µg), tobramycin (10 µg), amikacin (30 µg), ciprofloxacin (5 µg), trimethoprim-sulfamethoxazole (1.25/23.75 µg), tetracycline (30 µg), doxycycline (30 µg), chloramphenicol (30 µg), and fosfomycin (200 µg) (Becton Dickinson, Heidelberg, Germany). *E. coli* ATCC^®^ 25922 served as quality control strain for antimicrobial susceptibility testing. Extended-spectrum β-lactamase (ESBL) production by ESBL-test via agar disk diffusion was performed using cefotaxime and ceftazidime with and without clavulanic acid (Becton Dickinson, Heidelberg, Germany).

All *mcr*-*1* positive isolates underwent whole-genome sequencing (WGS). Isolation of DNA for WGS was performed with MagAttract HMW DNA Kit (Qiagen, Hilden, Germany). Nextera XT DNA Library Preparation Kit (Illumina, San Diego, CA, United States) was used for the preparation of ready-to-sequence libraries. Paired-end-sequencing of the investigated *E. coli* was done using the Illumina MiSeq platform with a read length of 2 × 300 bp [59]. SPAdes v.3.9.0 was used for de novo construction of raw reads [60]. SeqSphere+ software (Ridom, Münster, Germany) was used for WGS data analysis. Genetic relatedness of the investigated *mcr-1*-carrying isolates was assessed with MLST and cgMLST following a previously described protocol [61]. The *mcr-1*-carrying *E*. *coli* phylotypes and CH types were established from data obtained in WGS using Clermontyping (http://clermontyping.iame-research.center/, accessed on 1 August 2021) and using CHTyper hosted at Center for Genomic Epidemiology (https://cge.cbs.dtu.dk/services/chtyper/, accessed on 1 August 2021) [62,63,64]. Resistance genes and/or chromosomal mutations were identified with Comprehensive Antibiotic Resistance Database (CARD; https://card.mcmaster.ca/home, accessed on 1 August 2021) [65] as well as ResFinder 4.1 (https://cge.cbs.dtu.dk/services/ResFinder/, accessed on 1 August 2021) [66,67]. Genes encoding virulence factors were identified using VirulenceFinder 2.0 (https://cge.cbs.dtu.dk/services/VirulenceFinder/, accessed on 1 August 2021) as well as a database (http://www.mgc.ac.cn/VFs/, accessed on 1 August 2021) [68,69,70]. Serogenotypes were analyzed by SerotypeFinder (https://cge.cbs.dtu.dk/services/SerotypeFinder/, accessed on 1 August 2021) [71]. PlasmidFinder 2.1 (https://cge.cbs.dtu.dk/services/PlasmidFinder/, accessed on 1 August 2021) was used for the plasmid presence detection [72]. Probability prediction of the location of a *mcr* gene was achieved by applying mlplasmids trained on *E. coli* (accessed on 1 August 2021) [73]. The genomes of WGS isolates were deposited under PRJNA725684 in the NCBI BioProject database.

Biocides susceptibility testing was performed by broth microdilution as described by Schug et al., [74] using chlorhexidine (CHX, Sigma-Aldrich, Schnelldorf, Germany), benzalkonium chloride (BAC, Acros Organics, Geel, Belgium), glutardialdehyde (GLU, Chempur, Piekary Slaskie, Poland) and ispopropanol (ISO, 99.9%, PHPU Eurochem BGD, Tarnow, Poland). Concentration ranges were 0.000015–0.016% for BAC, 0.000015–0.002% for CHX, 0.0075–1% for GLU and 1–14% for ISO. *E. coli* ATCC^®^ 10536 (Microbiologics, St.Cloud, MN, USA) was tested for comparative reasons. This part of the investigation was performed at the Faculty of Biotechnology and Food Science in Wroclaw. Each biocide concentration was tested in 12 replicates. Testing was repeated three times at different intervals.

Ethical opinion and approval were not needed for this study due to the fact that clinical samples were obtained within the routine microbiological diagnostics and only subsequently evaluated within the scope of this study and therefore not subjected for reporting to the Ethics Commission for the experimental animals’ welfare protection of the Faculty of Veterinary Medicine in Belgrade, Serbia.

## 5. Conclusions

This is the first report of *mcr-1.1-*carrying *E. coli* from Serbia. Isolated strains belonged to ST410, ST58 and ST641 which were also previously never reported from Serbia, neither from humans nor animals. According to mlplasmids analyses, the *mcr-1.1* genes might be located on plasmids. Despite the *mcr-1* carrying *E. coli* were isolated from health animals, some of the detected virulence factors (*fyuA*, *fimH*, *hlyF*, *iss*, *ompT*, *sitA*, *traT*, *iroN*) are typical for ExPEC, therefore the pathogenic potential of these isolates and their significance for Public Health should not be excluded. All obtained MIC values of the tested biocides were in acceptable ranges, with no indication of possible resistance. Although only samples from turkeys were *mcr*-positive in this study, continuous monitoring of livestock samples is advised to prevent a spill-over from animals to humans.

## Figures and Tables

**Table 1:** Results of the molecular characterization and resistance profile of *mcr-1* carrying *E. coli* isolates.

Isolate ID	Phylogroup	Serotype	CH-	ST	CC		Resistance Profile	AA-Substitution	
						Phenotype	Genotype	GyrA	ParC
15/2 16/2 51	B1	O8:H25	CH4-32	ST58	CC155	AMP, FQR, TET, COL	*bla*_TEM-1_, *strA, strB, qrnS1, tet*(A), *mcr-1.1*	Ser83Leu	
52	B1	O29:H25	CH6-53	ST641	CC86	AMP, COL	*bla*_TEM-1_, *qrnS1*, *mcr-1.1*		
72/2	A	O8:H25	CH4-24	ST410	CC23	AMP, FQR, TET, SXT, CHL, COL,	*bla*_TEM-1_, *aadA1, aadA2, strA, strB, sul2, sul3, dfrA12, tet*(A), *tet*(M), *cmlA1*, *floR, mcr-1.1*	Ser83Leu, Asp87Asn	Ser80Ile

CH: clonotype; ST: sequence type; CC: clonal complex, AMP: ampicillin, CHL: chloramphenicol, COL: colistin, FQR: fluoroquinolone, SXT: trimethoprim/Sulfamethoxazole, TET: tetracycline.

**Table 2:** VAGs detected in the *mcr-1.1*-carrying *E. coli* isolates.

Isolate ID					VAG Class						
	Adherence	Secretion System	Iron Uptake	Autotransporter	TTSS Effectors	Invasion	Toxin	Antiphagocytosis	Motility	Protease	Serum Resistance
15/2, 16/2, 51	*cfaA, cfaB, cfaC, cfaD/cfaE, ecpA, ecpB, ecpC, ecpD, ecpE, elfA, elfC, elfD, elfG, eaeH, hcpA, hcpB, hcpC, fimA, fimC, fimD, fimE, fimF, fimG, fimH, fimI*	*aec15, aec16, aec17, aec18, aec19, aec22, aec23, aec24, aec25, aec26, aec27/clpV, aec28, aec29, aec30, aec31, aec32*	*sitA, sitB, sitC, sitD, iroB, iroC, iroD, iroE, iroN, fyuA, irp1, irp2, ybtA, ybtE, ybtP, ybtQ, ybtS, ybtT, ybtU, ybtX*	*cah, ehaA, ehaB, upaG/ehaG*	*espL1, espL4, espR1, espX1, espX4, espX5*	*ibeB, ibeC*	*hlyE/clyA*	*uge, wzc*	*flaA*	*ompT*	*rmlD*
52	*cfaA, cfaB, cfaC, cfaD/cfaE, ecpA, ecpB, ecpC, ecpD, ecp, elfA, elfC, elfD, elfG, eaeH, hcpA, hcpB, hcpC, fimA, fimC, fimD, fimE, fimF, fimG, fimH, fimI*	*ec15, aec16, aec17, aec18, aec19, aec22, aec23, aec24, aec25, aec26, aec27/clpV, aec28, aec29, aec30, aec31, aec32*	*sitA, sitB, sitC, sitD, iroB, iroC, iroD, iroE, iroN, fyuA, irp1, irp2. ybtA, ybtE, ybtP, ybtQ, ybtS, ybtT, ybtU, ybtX*	*cah, ehaA, ehaB, upaG/ehaG*	*espL1, espL4, espR1, espX1, espX4, espX5*	*ibeB, ibeC*	*hlyE/clyA*	*uge*	*flaA*		*rmlD*
72/2	*cfaA, cfaB, cfaC, cfaD/cfaE, ecpA, ecpB, ecpC, ecpD, ecpE, elfC, elfD, elfG, eaeH, hcpA, hcpB, hcpC, fimA, fimC, fimD, fimE, fimF, fimG, fimH, mrkB, mrkD*	*aec15, aec31, fliC*		*agn43, upaG/ehaG*	*espL1, espL4, espR1, espX5*	*ibeB, ibeC*	*hlyE/clyA*				

**Table 3:** Results of the biocide susceptibility testing of the five *mcr*-1-carrying *E. coli*.

Isolate ID	Biocide MIC Values [%]			
	Benzalkonium Chloride (BAC)	Chlorhexidine (CHX)	Isopropanol (ISO)	Glutardialdehyde (GDH)
**51**	0.001	0.00006	4	0.125
**52**	0.001	0.00003	6	0.125
**15/2**	0.001	0.00006	6	0.125
**16/2**	0.001	0.00006	4	0.125
**72/2**	0.002	0.00003	4	0.125

## Data Availability

All data are contained within the article.
